# Cost-Effectiveness of Adding SGLT2 Inhibitors to Standard Treatment for Heart Failure With Reduced Ejection Fraction Patients in China

**DOI:** 10.3389/fphar.2021.733681

**Published:** 2021-11-11

**Authors:** Yaohui Jiang, Rujie Zheng, Haiqiang Sang

**Affiliations:** Department Cardiology, The First Affiliated Hospital of Zhengzhou University, Zhengzhou, China

**Keywords:** dapagliflozin, empagliflozin, heart failure, cost-effectiveness analysis, China

## Abstract

**Objective:** To evaluate the economics and effectiveness of adding dapagliflozin or empagliflozin to the standard treatment for heart failure (HF) for patients with reduced ejection fraction (HFrEF) in China.

**Methods:** A Markov model was developed to project the clinical and economic outcomes of adding dapagliflozin or empagliflozin to the standard treatment for 66-year-old patients with HFrEF. A cost-utility analysis was performed based mostly on data from the empagliflozin outcome trial in patients with chronic heart failure and a reduced ejection fraction (EMPEROR-Reduced) study and the dapagliflozin and prevention of adverse outcomes in heart failure (DAPA-HF) trial. The primary outcomes were measured *via* total and incremental costs and quality-adjusted life years (QALYs) and the incremental cost-effectiveness ratio (ICER).

**Results:** In China, compared to the standard treatment, although adding dapagliflozin to the standard treatment in the treatment of HFrEF was more expensive ($4,870.68 vs. $3,596.25), it was more cost-effective (3.87 QALYs vs. 3.64 QALYs), resulting in an ICER of $5,541.00 per QALY. Similarly, adding empagliflozin was more expensive ($5,021.93 vs. $4,118.86) but more cost-effective (3.66 QALYs vs. 3.53 QALYs), resulting in an ICER of $6,946.69 per QALY. A sensitivity analysis demonstrated the robustness of the model in identifying cardiovascular death as a significant driver of cost-effectiveness. A probabilistic sensitivity analysis indicated that when the willingness-to-pay was $11,008.07 per QALY, the probability of the addition of dapagliflozin or empagliflozin being cost-effective was 70.5 and 55.2%, respectively. A scenario analysis showed that the cost of hospitalization, diabetes status, and time horizon had a greater impact on ICER.

**Conclusion:** Compared with standard treatments with or without empagliflozin, adding dapagliflozin to the standard treatment in the treatment of HFrEF in China was extremely cost-effective.

## Introduction

Heart failure (HF) is a serious clinical manifestation or a terminal stage of various heart diseases and has become an increasingly serious global public health problem (Conrad et al., 2018; Gu et al., 2003). In recent years, the prevalence of HF in China has increased to approximately 2%, and there are approximately 8–10 million patients experiencing HF (The US Centers for Disease Control and Prevention, 2016). It was estimated that the total direct and indirect costs related to HF in China in 2012 were approximately $0.84 billion (Cook et al., 2014), which would add a huge economic burden to China’s medical security system. Although great progress has been made in the field of HF treatment in the past 30 years, the 5-years mortality rate remains as high as 50%, and more than 50% of discharged patients will need to be hospitalized again within the next 6 months (Virani et al., 2020; Desai and Stevenson, 2012).

Sodium-glucose cotransporter 2 (SGLT2) inhibitors have been developed as a new therapeutic agent for the treatment of type 2 diabetes mellitus (T2DM), which can inhibit the proximal renal tubular SGLT protein family reabsorption of glucose, thereby reducing blood sugar levels (Chao and Henry, 2010). Notably there are some mechanisms pertaining to their cardiovascular (CV) benefits independently of blood glucose regulation, including natriuresis, increasing circulating ketone levels, anti-inflammatory effects, and reducing sympathetic overactivity (Andreadou et al., 2020; Iorga et al., 2020; Lymperopoulos et al., 2021). In particular, SGLT2 inhibitors better explain the left ventricle (LV) systolic function by improving cardiac energetics and reversing remodeling with reduction in LV volumes and LV mass (Garcia-Ropero et al., 2019; Jensen et al., 2020). SGLT2 inhibitors also improve LV diastolic function by reducing congestion and cardiac filling pressures (Santos-Gallego et al., 2021; Requena-Ibanez et al., 2021). Some studies have found that SGLT2 inhibitors regress interstitial myocardial fibrosis, reduce epicardial adipose tissue, and improve aortic stiffness (Nassif et al., 2021). The Empagliflozin Outcome Trial in Patients with Chronic Heart Failure and a Reduced Ejection Fraction (EMPEROR-Reduced) study and the Dapagliflozin and Prevention of Adverse Outcomes in Heart Failure (DAPA-HF) study found that both dapagliflozin and empagliflozin can reduce the risk of CV death or hospitalization in HFrEF patients with or without T2DM (Mcmurray et al., 2019; Packer et al., 2020). The Empagliflozin Outcome Trial in Patients with Chronic Heart Failure with Preserved Ejection Fraction (EMPEROR-Preserved) study found that empagliflozin could also be effective for heart failure with preserved ejection fraction (HFrEF) (Anker S. D. et al., 2021). Also, the United States Food and Drug Administration (FDA) announced that dapagliflozin and empagliflozin could be used for the treatment of HFrEF.

However, adding SGLT2 inhibitors to standard treatment in the treatment of HFrEF in China will significantly increase the cost of treatment. Several studies have been conducted in numerous European countries—including Thailand, Australia, and other countries to evaluate the cost-effectiveness of SGLT2 inhibitors for HFrEF (Mcewan et al., 2020; Savira et al., 2021; Krittayaphong and Permsuwan, 2021), but the medical systems and economic status of these countries are different from those of China. Therefore, it is essential to evaluate the economic impact among Chinese patients to guide clinicians and decision-makers to determine the best value of this new treatment option. Therefore, our study aimed to examine the cost-effectiveness of adding dapagliflozin or empagliflozin to the standard treatment of HFrEF in China.

## Methods and Material

### Module Building

**FIGURE 1:**
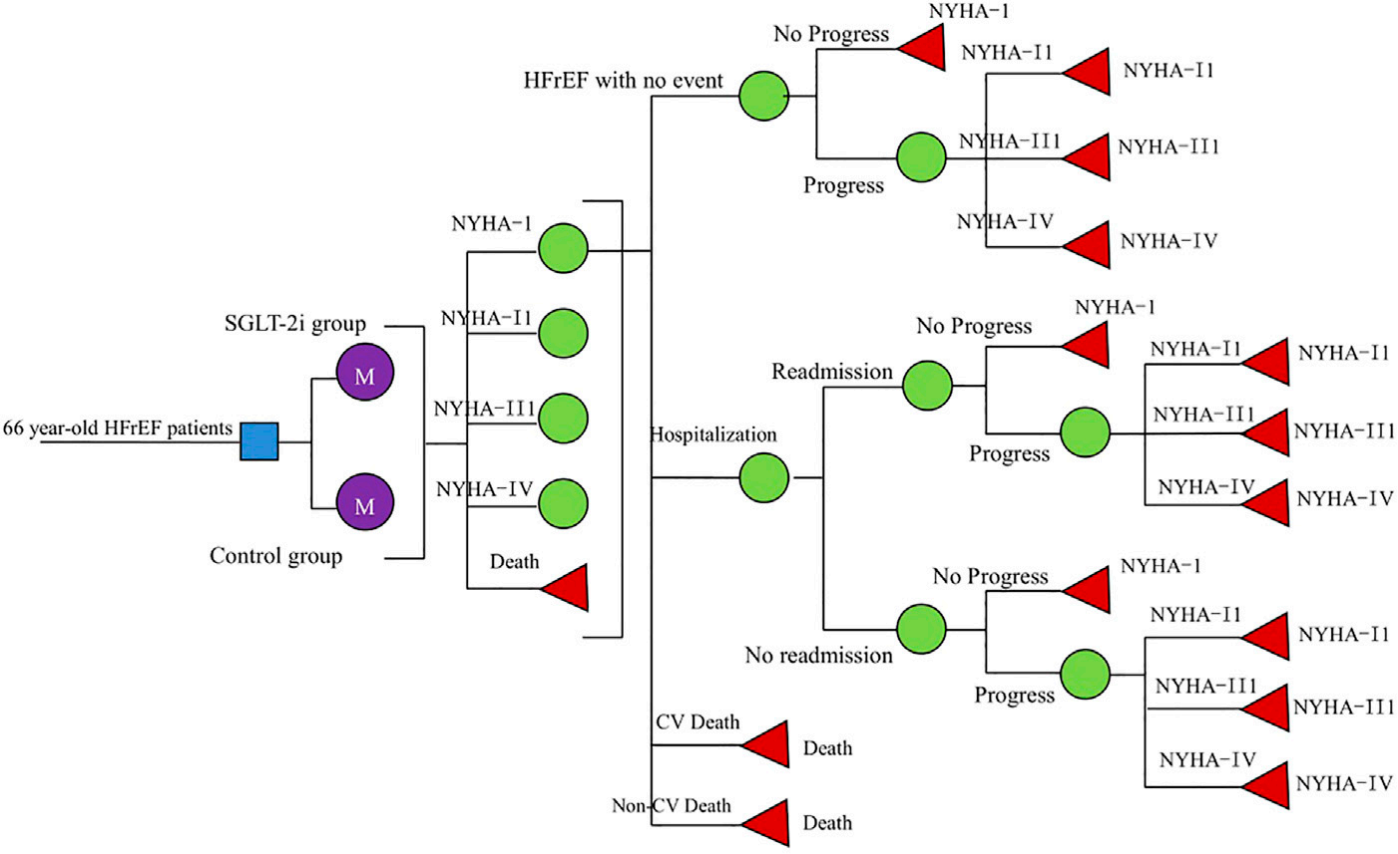
Schematic representation of the Markov model.

We constructed a Markov model for cost-utility analysis to compare the economics of three standard treatment options: standard treatment; adding dapagliflozin (10 mg, once daily) to the standard treatment; and adding empagliflozin (10 mg, once daily) to standard treatment. Based on the characteristics of the natural course of HFrEF and the availability of inter-state transition probability, this study set HFrEF patients into the following five states: New York Heart Association (NYHA) function classifications I, II, III, and IV and death, among which the death state was in the absorption state (Wu et al., 2020). Since the risk of readmission in the vulnerable period of HF was much higher than that in the stable period (Greene et al., 2015), we assumed that in our model, all patients who had experienced high-frequency hospitalizations had HF readmissions within 3 months. So, we arranged a fixed probability of readmission for each HF; at the end of each cycle, the patient switched between different NYHA function classifications. Events included hospitalization for HF, readmission for HF, CV death, and non-CV death. The patient can transfer between the states by pressing the arrow, as shown in Figure 1.

According to the EMPEROR-Reduced study and the DAPA-HF study, the inclusion criteria in our model were as follows: 1) age >18 years and diagnosis of HFrEF (NYHA II-IV) over 2 months; 2) LVEF ≤40% (LV ejection fraction) within the past 12 months; 3) N-terminal pro-brain natriuretic peptide (NT-proBNP) is elevated; and 4) receiving standard treatment for HFrEF, including drugs and medical devices. The exclusion criteria were as follows: 1) recently taking or tolerating SGLT2 inhibitors; 2) hypotension or systolic blood pressure below 95 mmHg; 3) type I diabetes; and 4) glomerular filtration rate (eGFR) < 30 ml/min/1.73 m^2^ (or rapid decline in renal function). The average age of the study population was 66 years. According to the natural outcome of the disease and the expected survival period of the population in this study, the model will be run for 10 years, with a period of 3 months (90 days), which is 40 cycles. According to the recommendations of the Chinese Pharmacoeconomic Evaluation Guide 2019 (Research Group of China Pharmacoeconomics Evaluation 2019), all costs and utilities ​​were discounted at an annual discount rate of 5%, and sensitivity analysis was performed between 0 and 8%. Our model used a half-period correction to prevent the overestimation of the expected survival time.

In the real world, the process of disease development, diagnosis, and treatment is more complicated, so some assumptions are needed in the model simulation to make the model reasonable and simplified. This study proposed the following hypotheses based on the progression of HFrEF and the process of diagnosis and treatment: 1) assuming that all patients were in stable HF before entering the long-term Markov model; 2) assuming that the effect of dapagliflozin and empagliflozin on HFrEF would not change with time; and 3) assuming that the probability of each event during 10 years would not be unchanged.

### Transition Probability

The initial NYHA function classification distribution in our cohort was derived from the DAPA-HF and the EMPEROR-Reduced studies (0% I, 71.3% II, 28% III, and 0.7% IV). In the DAPA-HF study, over the 18.2-months follow-up period, the rate of cardiovascular mortality (CM) in the dapagliflozin group and Control Group 1 was 9.6 and 11.5%, while the risk of hospitalization for HF was 9.7 and 13.4%,respectively (Mcmurray et al., 2019). During the EMPEROR-Reduced study’s 16 months follow-up period, the CM in the empagliflozin group was 10.0% and Control Group 2 was 10.8%, while the risk of hospitalization for HF in the empagliflozin group and Control Group 2 was 13.2 and 18.3%, respectively (Packer et al., 2020). Age-dependent non-CV deaths were all from the Report on China’s Cause of Death 2018, which is published by the China Center for disease Control and Prevention (National Center for Chronic and Noncommunicable Disease Control and Prevention, 2019). Furthermore, the readmission rate for HF was based on the literature published by Huang Jun (Huang et al., 2017). Based on the declining exponential approximation of life expectancy (DEALE) principle, the time length was converted into a rate, and then the rate was converted into a transition probability every 3 months (Park et al., 2019) with the following formula:

$$\text{r}=-\frac{1}{t}\text{ln}\left(S\right)$$

$$\text{P}=1-e^{-r*T}$$

Among them, S is the rate, t is the time, and P is the transition probability converted into every 3 months. We used the formula to calculate the transition probability of all parameters every 3 months (Table 1), and the 3-month transition probability between NYHA function classifications was also provided (King et al., 2016) (Table 2).

**TABLE 1:** Clinical input parameters.

Parameters	Value	Range	Distribution	Reference	Notes
Probability of CV mortality					
Dapagliflozin group	0.01650	0.01485–0.01815	Beta	Mcmurray et al. (2019)	±10% of the mean
Control1 group	0.01994	0.01795–0.02193	Beta	Mcmurray et al. (2019)	±10% of the mean
Empagliflozin group	0.01956	0.01760–0.02152	Beta	Packer et al. (2020)	±10% of the mean
Control2 group	0.02120	0.01908–0.02332	Beta	Packer et al. (2020)	±10% of the mean
Probability of HF hospitalization					
Dapagliflozin group	0.01668	0.0150–0.01835	Beta	Mcmurray et al. (2019)	±10% of the mean
Control1 group	0.02344	0.02110–0.02578	Beta	Mcmurray et al. (2019)	±10% of the mean
Empagliflozin group	0.02619	0.02357–0.02881	Beta	Packer et al. (2020)	±10% of the mean
Control2 group	0.03719	0.03347–0.04091	Beta	Packer et al. (2020)	±10% of the mean
Probability of non-CV mortality by age					
65–69 years	0.2430%			National Center for Chronic and Noncommunicable Disease Control and Prevention (2019)	Local data
70–74 years	0.3042%			National Center for Chronic and Noncommunicable Disease Control and Prevention (2019)	Local data
75–79 years	0.4185%			National Center for Chronic and Noncommunicable Disease Control and Prevention (2019)	Local data
Probability of HF readmission	0.1189	0.10701–0.13079	Beta	Huang et al. (2017)	±10% of the mean
Utility input					
NYHA I	0.2035	0.19525–0.2125	Beta	King et al. (2016)	95% CI
NYHA II	0.18	0.17325–0.18725	Beta	King et al. (2016)	95% CI
NYHA III	0.1475	0.13775–0.15725	Beta	King et al. (2016)	95% CI
NYHA IV	0.127	0.103–0.15125	Beta	King et al. (2016)	95% CI
Hospitalization and readmission	-0.1	-0.13–-0.08	Beta	King et al. (2016)	95% CI
Cost					
Standard treatment	$118.95	$118.95–556.21	Gammma	Huang et al. (2017)	95% CI
Dapagliflozin	$60.93	$48.74–73.12	Gammma	Local data	±20% of the mean
Empagliflozin	$ 59.25	$47.40–71.10	Gammma	Local data	±20% of the mean
Hospitalization and readmission	$1,785.36	$ 964.07–3209.47	Gammma	Ma, (2020)	Local data
Discounted rate	5%	0–8%		Research group of China Pharmacoeconomics Evaluation (2019)	

**TABLE 2:** New York Heart Association classification transition probabilities per cycle (3 months).

To	I	II	III	IV	Distribution
From					
I	0.977	0.019	0.004	0	Dirichlet
II	0.008	0.981	0.010	0.001	Dirichlet
III	0	0.034	0.960	0.006	Dirichlet
IV	0	0	0.055	0.945	Dirichlet

### Cost

From the perspective of the Chinese medical and health system, this study only calculated direct medical costs. The standard treatment cost included angiotensin converting enzyme inhibitors (ACEI), angiotensin receptor blockers (ARBs), beta-blockers, spironolactone, and diuretics from a previous study (Huang et al., 2017). Moreover, we assumed that the standard treatment was $102.75 per cycle, which was converted to $118.95 in 2020 according to the annual discount rate of 5%. Considering that approximately 10% of the DAPA-HF study took sacubitril/valsartan (SAC/VAL) and 19% of the EMPEROR-Reduced study took SAC/VAL, we calculated that the cost of SAC/VAL for 3 months was $556.21 (target dose 200 mg, twice daily). Correspondingly, according to the latest national negotiation price in 2020, enalapril was $0.087 per 10 mg twice daily and SAC/VAL was $3.10 per 200 mg twice daily, so the range of standard treatment costs was calculated (Table 1). The cost of hospitalization for HF was from the China Health Statistics Yearbook 2020, which included town-level, county-level, municipal, provincial, and ministerial hospitals. We calculated that hospitalization cost $1,785.36 (Ma, 2020), dapagliflozin was $0.677 per 10 mg daily, and empagliflozin was $0.658 per 10 mg daily according to the latest national negotiation price in 2020; also, the 90-days cost was $60.93 for dapagliflozin and $59.25 for empagliflozin (Table 1). All costs were converted at the rate of.

6.44 ¥/USD (The People’s Bank of China, 2020).

### Utility

In this study, quality-adjusted life years (QALYs) were used as a measure of effect. The utility of different levels of NYHA function classifications was derived from published literature (Table 1), and scores were based on a scale from 0 (death) to 1 (perfect health). NYHA I through IV used a one-time utility of −0.1, for each hospitalization and readmission event (Table 1) (King et al., 2016).

### Outcome

The primary endpoints in this study were QALY, cost, and the incremental cost-effectiveness ratio (ICER). Notably, the following is according to the recommendation of the World Health Organization (WHO) for the evaluation of pharmacoeconomics (Eichler et al., 2004): ICER <1 fold of gross domestic product (GDP) per capita, the increased cost is completely worth it and very cost-effective; 1 fold of GDP per capita < ICER <3 fold of GDP per capita, the increased cost is acceptable and cost-effective; ICER >3 fold of GDP per capita, the increased cost is not worth it and not cost-effective. According to the data released by the National Bureau of Statistics, per capita GDP in 2019 in China was $11,008.07 (National Bureau of Statistics of the People’s Republic of China, 2019). Given this, we used one time per capita GDP ($11,008.07 per QALY) in 2019 as the threshold standard and the willingness-to-pay (WTP) to judge whether a health intervention is cost-effective.

### Sensitivity Analysis

One-way sensitivity was performed to investigate the effects of uncertainty in the model. The model parameters were varied over 95% confidence intervals. Variations of ±10% and ±20% were assumed for parameters of probability and medical costs that have no specified data range (Table 1), and the results of each parameter on the ICER are displayed as a tornado diagram.

This study also performed a scenario analysis of diabetes status, hospitalization costs, and time horizon. According to the DAPA-HF and the EMPEROR-Reduced studies, for the non-diabetic and diabetic subgroups, the CM or rehospitalization for HF in the dapagliflozin group or empagliflozin group was lower than that in the control group (Petrie et al., 2020; Anker SD. et al., 2021). There were different levels of hospitals, including town-level hospitals ($964.07); county-level hospitals ($1,120.75); municipal hospitals ($1,785.36); provincial hospitals ($2,812.51); and ministerial hospitals ($3,209.47). The time horizon of 5, 10, 15, and 20 years was also changed to explore its impact on the estimated ICER.

A probabilistic sensitivity analyses (PSA) was also carried out to investigate the uncertainty of all the parameters simultaneously. We assumed that the cost followed the gamma distribution and the utility and the transition probability followed the beta distribution. This was achieved by calculating the results of 1,000 Monte Carlo simulations with different parameter distributions, which were transformed into cost-effectiveness acceptability curves (CEACs).

## Results

### Model Validation and Clinical Results

The average age of the simulated population in this study was 66 years. Our model predicted that the all-cause mortality at 18 months in the dapagliflozin group was 10.9%, the CM was 9.08%, and the rate of hospitalization for HF was 11.0.%; the all-cause mortality in Control Group 1 was 12.8%, the CM was 10.98%, and the rate of hospitalization for HF was 15.3%; the all-cause mortality at 16 months in the empagliflozin group was 11.6%, the CM was 10.04%, and the rate of hospitalization for HF was 15.5%; the all-cause mortality in Control Group 2 was 12.4%, the CM was 10.84%, and the rate of hospitalization for HF was 22.5%. The median survival time of the dapagliflozin group and Control Group 1 was 8.75 and 7.50 years, respectively; the median survival time of the empagliflozin group and Control Group 2 were 7.5 and 7.25 years, respectively. These median survival times indicated to us that the outcome predicted by our model was close to the results of clinical trials.

### Cost-Utility Analysis

The results are presented in Table 3. The total utility of the dapagliflozin group after 40 cycles was 3.87 QALYs, which was 0.23 QALYs higher than Control Group 1. The total cost of the dapagliflozin group was $4,870.68, which was $1,274.43 higher than Control Group 1, and the ICER was $5,541.00 per QALY, which was lower than China’s per capita GDP of $11,008.07 in 2019. So, this indicated that the dapagliflozin group was more cost-effective. The total utility of the empagliflozin group after 40 cycles was 3.66 QALYs, which was 0.13 QALYs higher than that of Control Group 2 and the total cost of the empagliflozin group was $5,021.93, which was $903.07 higher than that of Control Group 2. Furthermore, the ICER was 6,946.69 per QALY, which was lower than China’s per capita GDP of $11,008.07 in 2019. Accordingly, the empagliflozin group was more cost-effective and so the dapagliflozin group had an absolute economic advantage compared with the empagliflozin group.

**TABLE 3:** The results from base-case analysis.

	Total cost ($)	Total life years (QALY)	Incremental cost ($)	Incremental life years (QALY)	ICER($ per QALY)
Dapagliflozin group	4,870.68	3.87	1,274.43	0.23	5,541.00
Control1 group	3,596.25	3.64			
Empagliflozin group	5,021.93	3.66	903.07	0.13	6,946.69
Control2 group	4,118.86	3.53			

### Sensitivity Analysis

**FIGURE 2:**
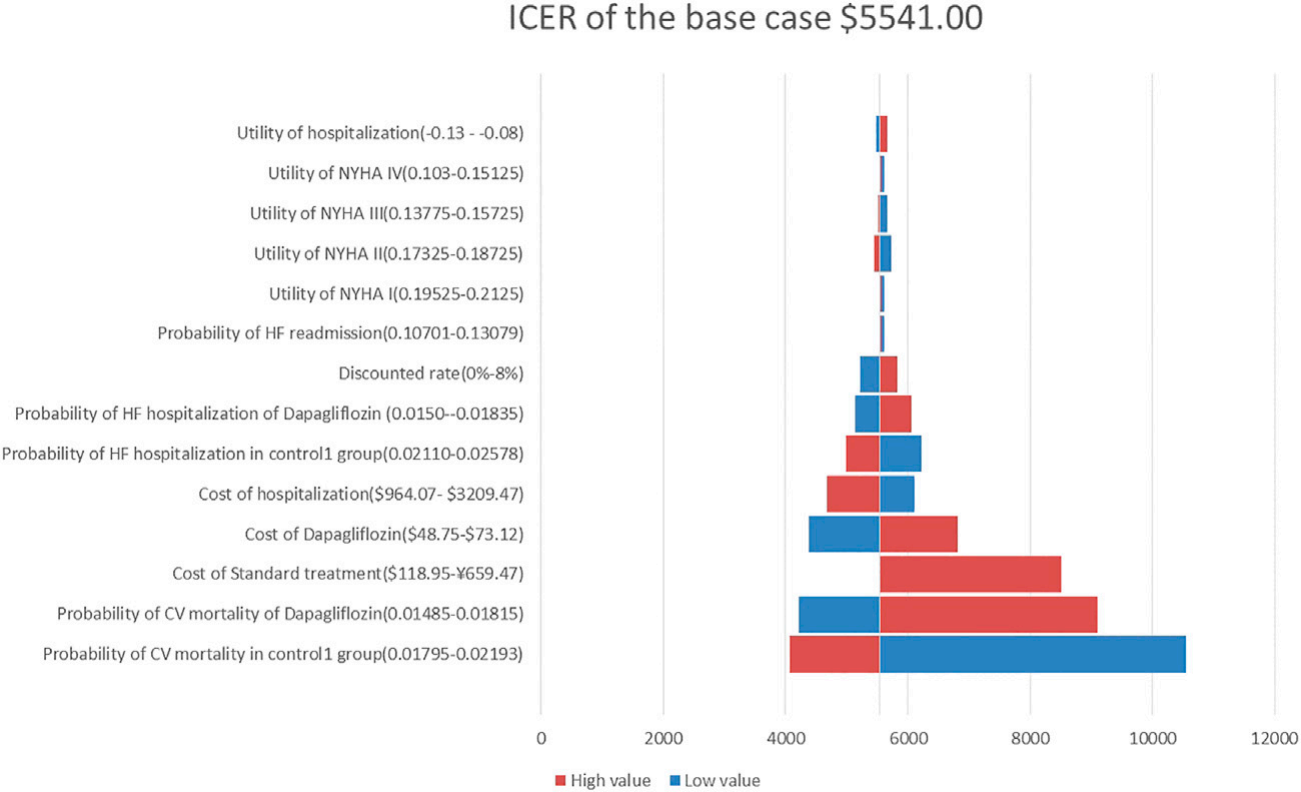
Tornado diagram showing the univariate sensitivity analysis of the Markov model simulation (Dapagliflozin group vs. Control group 1).

**FIGURE 3:**
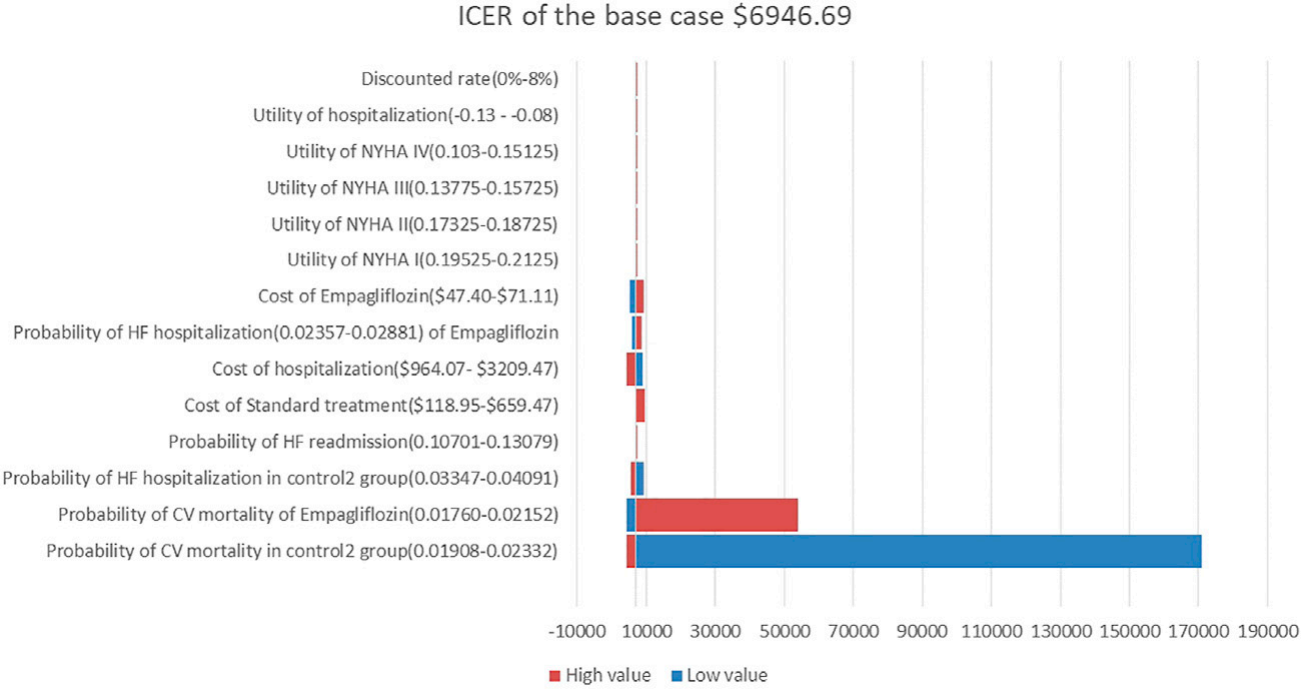
Tornado diagram showing the univariate sensitivity analysis of the Markov model simulation. (Empagliflozin group vs. Control group 2).

A one-way sensitivity analysis of the dapagliflozin group and Control Group 1 is shown in Figure 2. When all parameters changed within the set range of variation, the ICER was within 1 fold per capita GDP, and the one-way sensitivity analysis of the empagliflozin group and Control Group 2 are shown in Figure 3. The low value of CM in Control Group 2 and the high value of CM in the empagliflozin group had a greater impact on the results, which was far more than one fold per capita GDP, but other parameters had little impact.

Based on the scenario analysis, in both the dapagliflozin and empagliflozin groups, the ICER of the diabetic group was lower than that of the non-diabetic group; as the cost of hospitalization for different levels of hospitals increased, the ICER gradually decreased, and as the time horizon became longer, the ICER gradually decreased (see Table 4).

**TABLE 4:** The result of scenario analyses presented as ICER.

Scenario	Dapagliflozin	Empagliflozin
	ICER(($ per QALY))	ICER(($per QALY))
Diabetes		
With	4,411.18	5,016.44
Without	6,790.06	10,844.36
Hospital characteristic		
Town Hospital	6,113.96	8,852.76
County Hospital	6,013.99	8,538.35
Municipal Hospital	5,589.93	7,204.65
Provincial Hospital	5,558.75	7,106.52
Ministerial Hospital	4,681.28	4,346.83
Time horizon		
5 years	8,493.52	9,975.67
10 years	5,589.93	7,204.65
15 years	4,600.59	5,359.84
20 years	4,151.68	5,077.71

**FIGURE 4:**
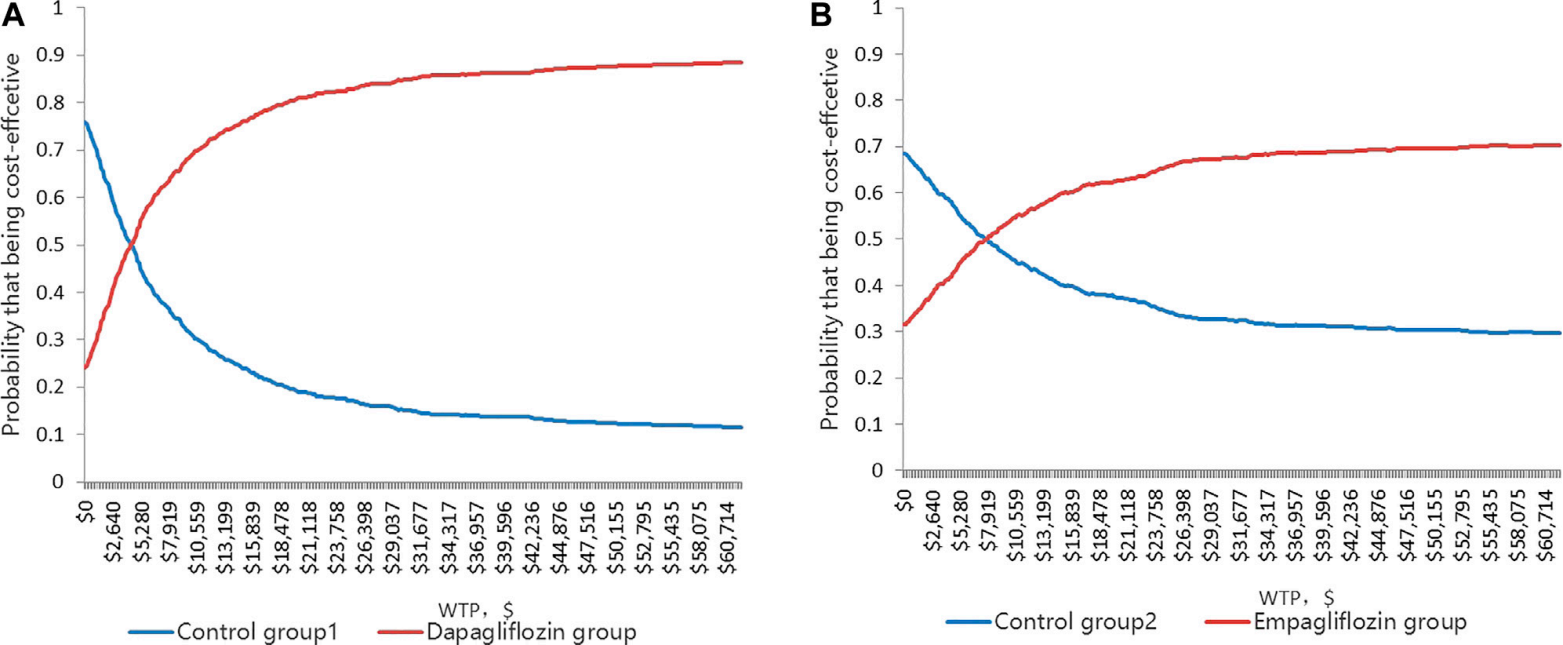
**(A)** Cost-effectiveness acceptability curve showing the maximum willingness to pay and the corresponding probability of cost-effectiveness for Dapagliflozin group and Control group 1. **(B)** Cost-effectiveness acceptability curve showing the maximum willingness to pay and the corresponding probability of cost-effectiveness for Empagliflozin group and Control group 2.

**FIGURE 5:**
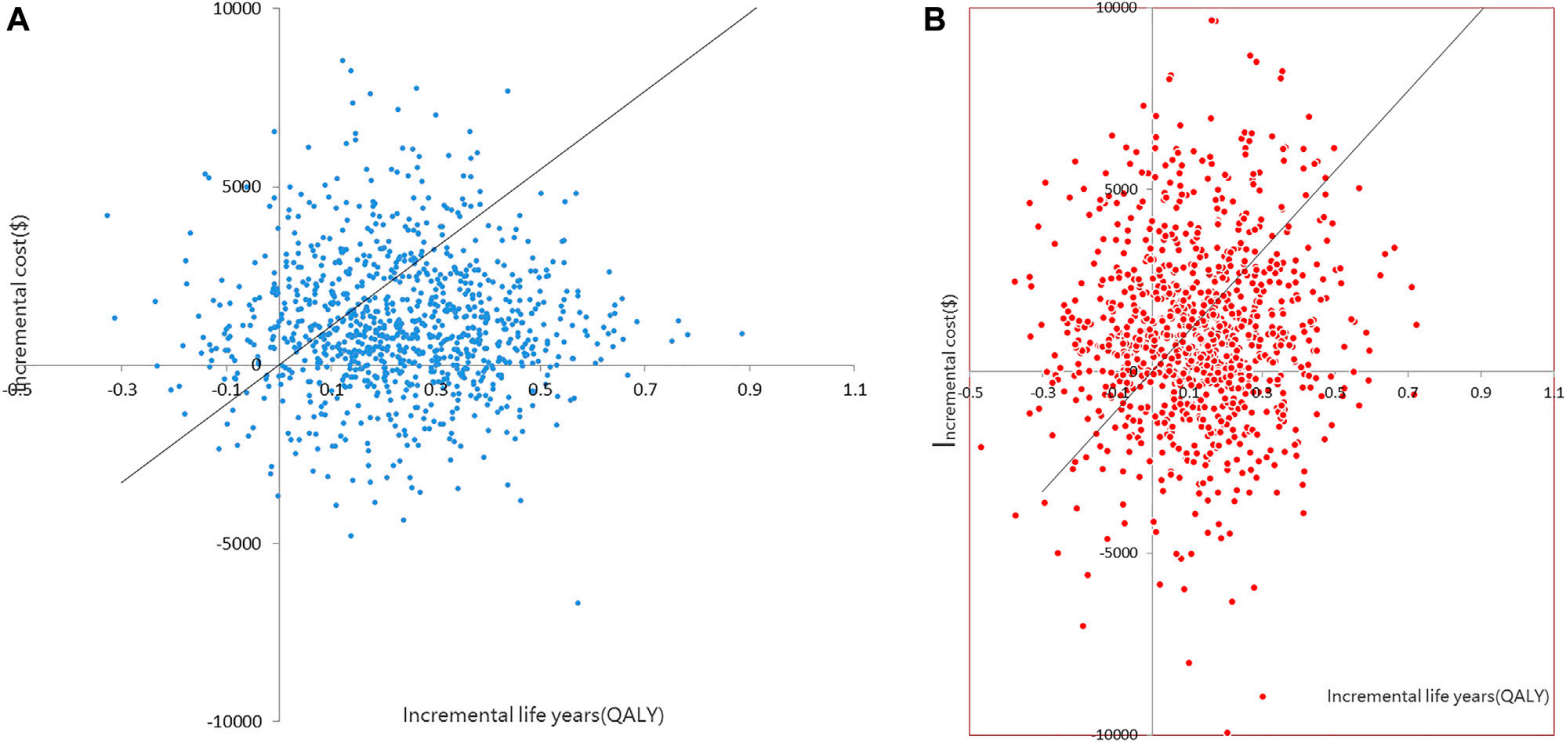
**(A)** Scatter plot showing the incremental costs and incremental quality-adjusted life-year of a thousand simulations for Dapagliflozin group and Control group 1. **(B)** Scatter plot showing the incremental costs and incremental quality-adjusted life-year of a thousand simulations for Empagliflozin group and Control group 2).

The CEACs (Figures 4A,B) were shown when the WTP was $11,008.07, and the probability that the dapagliflozin and empagliflozin groups were 70.5 and 55.2%, respectively. The results of the PSA based on 1,000 Monte Carlo simulations are presented as a scatter plot (Figures 5A,B) where the scattered points were mainly distributed in the first quadrant and most of them were below the WTP threshold line. The PSA results were similar to the basic analysis results; the dapagliflozin group was more cost-effective.

## Discussion

This study is the first cost-utility study to add dapagliflozin or empagliflozin to the standard treatment in the treatment of HFrEF in China based on data from the EMPEROR-Reduced and the DAPA-HF studies—as well as China’s public databases. Our study showed that compared with standard treatments with or without empagliflozin, adding dapagliflozin to the standard treatment in the treatment of HFrEF in China was extremely cost-effective. The ICER was $5,541 per QALY, which was lower than China’s per capita GDP of $11,008.07 in 2019. According to our model, it is assumed that 10 million HF patients will be treated with dapagliflozin in the standard treatment, which reduces 300,000 hospitalizations for HF and 180,000 deaths. The medical cost of hospitalization for HF will save $1.4 billion, greatly reducing the burden on China’s medical security system. There is a huge base of 8–10 million HF patients in China (The US Centers for Disease Control and Prevention, 2016), and up to half of them are HFrEF. Therefore, adding dapagliflozin to the standard treatment can reduce medical costs and improve the prognosis of HFrEF. Overall, our results provide decision-makers and healthcare payers with a valuable quantitative assessment of dapagliflozin.

In our one-way sensitivity analysis, it was found that CM in the dapagliflozin group and Control Group 1 had a great impact on the ICER, but the ICER was less than 1 fold per capita GDP, indicating that our model was stable and reliable. The CM in the empagliflozin group and Control Group 2 had a great impact on the ICER value, which was far more than 1 fold per capita GDP. We believe that this is due to the results of the EMPEROR-Reduced study that empagliflozin cannot reduce the risk of CM in patients with HFrEF (hazard ratio, 0.92; 95% CI, 0.75–1.12) (Mcmurray et al., 2019). If the range of this parameter is changed, the ICER will change significantly, which cannot be considered as the result of model instability. Whether adding dapagliflozin or empagliflozin to the standard treatment is cost-effective is mainly dependent on the clinical effects on the HFrEF patients, including reducing the risk of CM and the risk of hospitalization for HF.

In the PSA, it was found that the probability of adding dapagliflozin or empagliflozin was lower than that in other similar studies on HF (Savira et al., 2021; Van der Pol et al., 2017), whose probability was often more than 90%. This is because the medical system and economic status of these countries were different from those of China. The cost of hospitalization was $10,000, and the WTP ranged from $30,000 to $50,000. The scenario analysis also proved that the higher the cost of hospitalization, the more cost-effective it was.

Diabetes is closely related to HF, and it is estimated that 10% of diabetic patients suffer from HF (Bank et al., 2017). In fact, HF is the second most common CV manifestation of diabetes, and the prognosis of HF in diabetic patients is worse than that in non-diabetic patients (Bank et al., 2017; Shah et al., 2015; Jia et al., 2018). The DAPA-HF subgroup analysis showed that dapagliflozin reduces the risk of CV deaths by 15 and 21% in non-diabetic and diabetic populations, respectively (Petrie et al., 2020). Furthermore, dapagliflozin significantly reduced in people of varied ages (<55 years old, 55–64 years old, 65–74 years old, ≥ 75 years old) the risk of a CV death or an HF worsening by 13, 29, 24, and 32%, respectively (Martinez et al., 2020). In the scenario analysis, we also found that the ICER of the diabetic population was lower, and the longer the time of adding dapagliflozin to the standard treatment, the more cost-effective it was. Moreover, in China, compared with metformin and glimepiride, dapagliflozin was cost-effective in treating T2DM (Cai et al, 2019; Gu et al., 2016; Shao et al., 2017). Also, for patients with HFrEF and T2DM in China, adding dapagliflozin to their standard treatment not only greatly reduces the cost of medication and hospitalization, but is also more cost-effective.

In addition, the DAPA-HF study found that dapagliflozin could reduce the risk of CV death and hospitalization for HF in patients with HFrEF by 18 and 30%, respectively (Mcmurray et al., 2019), while the Prospective Comparison of ARNI with ACEI to Determine Impact on Global Mortality and Morbidity in Heart Failure (PARADIGM-HF) study showed that compared with enalapril, SAC/VAL could reduce the risk of CV deaths and hospitalization for HF in patients with HFrEF by 20 and 21%, respectively (Mcmurray et al., 2014). Although there is no prospective study comparing the effects of dapagliflozin and SAC/VAL in the treatment of HFrEF, the results of clinical trials are similar. According to the latest national negotiation price in 2020, SAC/VAL is $3.5 per 200 mg twice daily, the daily cost is about $6.18 in the PARADIGM-HF study; dapagliflozin is $0.677 per 10 mg daily, the daily cost is about $0.677 for low-income patients; and dapagliflozin is the first-choice drug.

There were some limitations in this study. First, our model did not consider hospitalization for non-HF, but in the DAPA-HF and EMPEROR-Reduced studies, the hazard ratio of all-cause hospitalization was 0.75 and 0.85, respectively (Mcmurray et al., 2019; Packer et al., 2020), and our results could be conservative. Second, we could not obtain data regarding dapagliflozin and empagliflozin in patients with HFrEF in China and the health utility of each state, which may lead to some racial bias in the simulation results. Third, we assumed that HF patients in China could tolerate the recommended dose of each drug, regardless of the adverse events. In the DAPA-HF and EMPEROR-Reduced studies, the most common adverse events including hypovolemia, renal failure, amputation, diabetic ketoacidosis, and gangrene were not significantly different. Fourth, other possible real-world treatment strategies were not calculated, such as drug switching, drug compliance heart transplantation, etc. Finally, in our model, the transition probability is fixed, which is not calculated by age distribution, but as the age becomes older, the clinical benefit of dapagliflozin is higher (Martinez et al., 2020), and the ICER is smaller, which further emphasizes that the results of the analysis may be conservative.

## Conclusion

In conclusion, our analysis provided an insight into the cost-effectiveness of adding dapagliflozin or empagliflozin in treating HFrEF patients compared with only the standard treatment. Adding dapagliflozin was considered cost-effective based on the perspective of the Chinese public healthcare system. Accordingly, our findings will help healthcare providers make decisions. Additional real-world studies on the cost-effectiveness of dapagliflozin or empagliflozin based on the Chinese population need to be conducted.

## Data Availability

The original contributions presented in the study are included in the article/Supplementary Material, further inquiries can be directed to the corresponding author.
